# 1-[(*Z*)-2-Butyl­tellan­yl-1-chloro­ethen­yl]­cyclo­hex-1-ene

**DOI:** 10.1107/S1600536812007490

**Published:** 2012-02-24

**Authors:** Julio Zukerman-Schpector, Ignez Caracelli, Rafael Carlos Guadagnin, Hélio A. Stefani, Edward R. T. Tiekink

**Affiliations:** a Departmento de Química, Universidade Federal de São Carlos, CP 676, 13565-905 São Carlos, SP, Brazil; b BioMat – Departmento de Física, Universidade Federal de São Carlos, CP 676, 13565-905 São Carlos, SP, Brazil; c Departamento de Ciências Exatas e da Terra, Universidade Federal de São Paulo – Campus Diadema, Rua Professor Artur Ridel, 275, 09972-270 Diadema, SP, Brazil; d Departamento de Farmácia, Faculdade de Ciências Farmacêuticas, Universidade de São Paulo, São Paulo, SP, Brazil; e Department of Chemistry, University of Malaya, 50603 Kuala Lumpur, Malaysia

## Abstract

The Te^II^ atom in the title mol­ecule, C_12_H_19_ClTe, is coordinated in a V-shaped geometry by C atoms derived from the disparate organic substituents. A short intramolecular C—H⋯Cl contact occurs owing to the proximity of the ethene bond in the six-membered ring to the Cl atom. In the crystal, mol­ecules assemble into layers parallel to the *ac* plane, with the closest inter­actions between them being of the Te⋯Te type [3.9993 (16) Å].

## Related literature

For background to the synthesis, see: Guadagnin *et al.* (2008 ▶). For related crystal structures, see: Zeni *et al.* (1999 ▶); Barrientos-Astigarraga *et al.* (2002 ▶). For ring conformational analysis, see: Cremer & Pople (1975 ▶). The van der Waals radius for Te was taken from Bondi (1964 ▶).
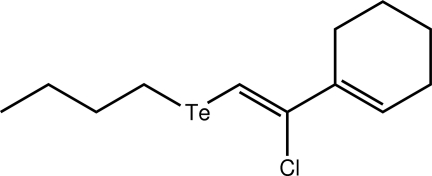


## Experimental

### 

#### Crystal data

C_12_H_19_ClTe
*M* *_r_* = 326.32
Triclinic, 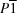

*a* = 7.666 (3) Å
*b* = 7.687 (3) Å
*c* = 12.266 (4) Å
α = 95.499 (15)°
β = 105.060 (14)°
γ = 111.832 (13)°
*V* = 632.8 (4) Å^3^
*Z* = 2
Mo *K*α radiation
μ = 2.53 mm^−1^
*T* = 100 K
0.3 × 0.3 × 0.2 mm

#### Data collection

Rigaku Saturn724 (2 × 2 bin mode) diffractometer
Absorption correction: multi-scan (*ABSCOR*; Higashi, 1995 ▶) *T*_min_ = 0.575, *T*_max_ = 1.000
3588 measured reflections
2421 independent reflections
2406 reflections with *I* > 2σ(*I*)
*R*_int_ = 0.024

#### Refinement

*R*[*F* ^2^ > 2σ(*F* ^2^)] = 0.023
*wR*(*F* ^2^) = 0.069
*S* = 1.17
2421 reflections
128 parameters
H-atom parameters constrained
Δρ_max_ = 0.86 e Å^−3^
Δρ_min_ = −0.95 e Å^−3^

### 

Data collection: *CrystalClear* (Molecular Structure Corporation & Rigaku, 2005 ▶); cell refinement: *CrystalClear*; data reduction: *CrystalClear*; program(s) used to solve structure: *SIR97* (Altomare *et al.*, 1999 ▶); program(s) used to refine structure: *SHELXL97* (Sheldrick, 2008 ▶); molecular graphics: *ORTEP-3* (Farrugia, 1997 ▶) and *DIAMOND* (Brandenburg, 2006 ▶); software used to prepare material for publication: *MarvinSketch* (ChemAxon, 2010 ▶) and *publCIF* (Westrip, 2010 ▶).

## Supplementary Material

Crystal structure: contains datablock(s) global, I. DOI: 10.1107/S1600536812007490/zl2453sup1.cif

Structure factors: contains datablock(s) I. DOI: 10.1107/S1600536812007490/zl2453Isup2.hkl

Supplementary material file. DOI: 10.1107/S1600536812007490/zl2453Isup3.cml

Additional supplementary materials:  crystallographic information; 3D view; checkCIF report

## Figures and Tables

**Table:** 

Te—C1	2.077 (3)
Te—C9	2.148 (3)

**Table:** 

C1—Te—C9	94.09 (12)

**Table 2:** Hydrogen-bond geometry (Å, °)

*D*—H⋯*A*	*D*—H	H⋯*A*	*D*⋯*A*	*D*—H⋯*A*
C8—H8⋯Cl	0.95	2.56	3.024 (3)	110

**Fig. 1.**
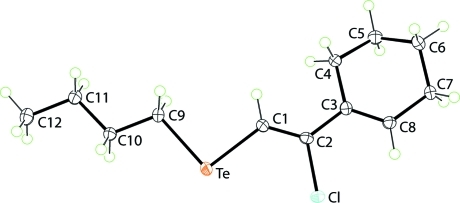
The molecular structure of (I) showing the atom-labelling scheme and displacement ellipsoids at the 50% probability level.

**Fig. 2.**
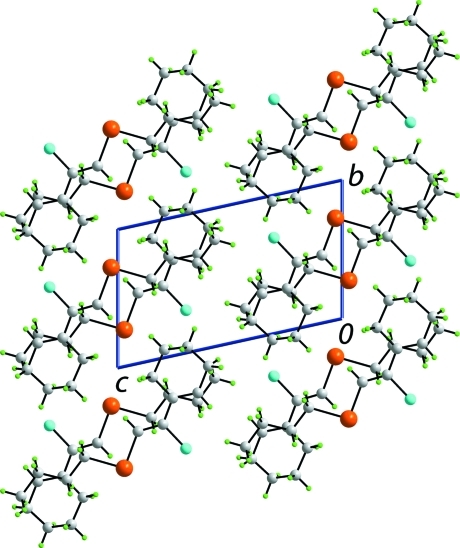
A view in projection down the a axis of the unit-cell contents for (I).

## supplementary crystallographic information

### Comment

The title compound, (I), was synthesized by reduction with sodium borohydride of the corresponding 2-halovinyl tellurium dichloride (Guadagnin *et al.*, 2008).

The Te^II^ atom in (I), Fig. 1, is coordinated by C atoms derived from the organic substituents which define a V-shape, Table 1. The conformation about the C1═C2 bond [1.334 (4) Å] is *Z*. The ethene bond in the six-membered ring is orientated toward the Cl atom enabling the formation of an intramolecular C—H···Cl interaction, Table 2. The conformation of the six-membered ring is a half-chair with the C5 atom lying 0.641 (5) Å above the plane of the remaining five atoms (r.m.s. deviation = 0.0738 Å), with puckering parameters: q_2_ = 0.381 (4) Å and q_3_ = 0.311 (4) Å, and amplitudes: Q = 0.492 (4) Å, θ = 50.8 (5) ° and φ_2_ = 144.3 (6) ° (Cremer & Pople, 1975).

In the crystal packing, molecules assemble into layers parallel to the *ac* plane, Fig. 2. Within layers, Te···Te^i^ contacts of 3.9993 (16) Å, *i.e.* less than the sum of the van der Waals radius for Te of 4.4 Å (Bondi, 1964), are noted; *i*: -*x*, -*y*, 2 - *z*.

### Experimental

The title compound was prepared as described in a previous study (Guadagnin *et al.*, 2008). Crystals of (I) were obtained by slow evaporation from its CHCl_3_ solution held at room temperature.

### Refinement

C-bound H-atoms were placed in calculated positions (C—H = 0.95–0.99 Å) and were included in the refinement in the riding model approximation with *U*_iso_(H) = 1.2–1.5*U*_eq_(C). Owing to poor agreement, three reflections, *i.e.* (2 8 3), (1 4 4) and (1 5 6), were omitted from the final refinement.

### Crystal data

**Table:**

C_12_H_19_ClTe	*Z* = 2
*M**_r_* = 326.32	*F*(000) = 320
Triclinic, *P*1	*D*_x_ = 1.713 Mg m^−^^3^
Hall symbol: -P 1	Mo *K*α radiation, λ = 0.71073 Å
*a* = 7.666 (3) Å	Cell parameters from 8499 reflections
*b* = 7.687 (3) Å	θ = 1.7–29.8°
*c* = 12.266 (4) Å	µ = 2.53 mm^−^^1^
α = 95.499 (15)°	*T* = 100 K
β = 105.060 (14)°	Irregular, yellow
γ = 111.832 (13)°	0.3 × 0.3 × 0.2 mm
*V* = 632.8 (4) Å^3^	

### Data collection

**Table:**

Rigaku Saturn724 (2x2 bin mode) diffractometer	2421 independent reflections
Radiation source: fine-focus sealed tube	2406 reflections with *I* > 2σ(*I*)
Graphite monochromator	*R*_int_ = 0.024
Detector resolution: 28.5714 pixels mm^-1^	θ_max_ = 26.0°, θ_min_ = 2.9°
ω scans	*h* = −8→9
Absorption correction: multi-scan (*ABSCOR*; Higashi, 1995)	*k* = −9→7
*T*_min_ = 0.575, *T*_max_ = 1.000	*l* = −15→14
3588 measured reflections	

### Refinement

**Table:**

Refinement on *F*^2^	Primary atom site location: structure-invariant direct methods
Least-squares matrix: full	Secondary atom site location: difference Fourier map
*R*[*F*^2^ > 2σ(*F*^2^)] = 0.023	Hydrogen site location: inferred from neighbouring sites
*wR*(*F*^2^) = 0.069	H-atom parameters constrained
*S* = 1.17	*w* = 1/[σ^2^(*F*_o_^2^) + (0.0343*P*)^2^ + 1.4833*P*] where *P* = (*F*_o_^2^ + 2*F*_c_^2^)/3
2421 reflections	(Δ/σ)_max_ < 0.001
128 parameters	Δρ_max_ = 0.86 e Å^−^^3^
0 restraints	Δρ_min_ = −0.95 e Å^−^^3^

### Special details

**Table:**

Geometry. All s.u.'s (except the s.u. in the dihedral angle between two l.s. planes) are estimated using the full covariance matrix. The cell s.u.'s are taken into account individually in the estimation of s.u.'s in distances, angles and torsion angles; correlations between s.u.'s in cell parameters are only used when they are defined by crystal symmetry. An approximate (isotropic) treatment of cell s.u.'s is used for estimating s.u.'s involving l.s. planes.
Refinement. Refinement of *F*^2^ against ALL reflections. The weighted *R*-factor *wR* and goodness of fit *S* are based on *F*^2^, conventional *R*-factors *R* are based on *F*, with *F* set to zero for negative *F*^2^. The threshold expression of *F*^2^ > 2σ(*F*^2^) is used only for calculating *R*-factors(gt) *etc*. and is not relevant to the choice of reflections for refinement. *R*-factors based on *F*^2^ are statistically about twice as large as those based on *F*, and *R*- factors based on ALL data will be even larger.

### Fractional atomic coordinates and isotropic or equivalent isotropic displacement parameters (Å^2^)

**Table:**

	*x*	*y*	*z*	*U*_iso_*/*U*_eq_	
Te	0.10592 (3)	0.26493 (3)	0.970657 (16)	0.01857 (9)	
Cl	0.04726 (12)	0.30822 (11)	0.69597 (6)	0.02053 (17)	
C10	0.1785 (5)	0.2722 (4)	1.2266 (3)	0.0163 (6)	
H10A	0.2188	0.1662	1.2121	0.020*	
H10B	0.0323	0.2162	1.2066	0.020*	
C2	0.2010 (4)	0.5213 (4)	0.7990 (3)	0.0143 (6)	
C4	0.3968 (5)	0.8795 (4)	0.8452 (3)	0.0171 (6)	
H4A	0.5292	0.8862	0.8866	0.020*	
H4B	0.3262	0.8801	0.9022	0.020*	
C3	0.2828 (4)	0.6957 (4)	0.7553 (3)	0.0140 (6)	
C6	0.4941 (5)	1.0420 (5)	0.6892 (3)	0.0213 (7)	
H6A	0.5152	1.1599	0.6582	0.026*	
H6B	0.6223	1.0306	0.7137	0.026*	
C11	0.2714 (5)	0.3691 (5)	1.3547 (3)	0.0190 (6)	
H11A	0.4177	0.4269	1.3747	0.023*	
H11B	0.2292	0.4736	1.3698	0.023*	
C9	0.2398 (5)	0.4113 (5)	1.1492 (3)	0.0184 (6)	
H9A	0.3860	0.4681	1.1688	0.022*	
H9B	0.1980	0.5167	1.1623	0.022*	
C5	0.4221 (5)	1.0565 (5)	0.7922 (3)	0.0218 (7)	
H5A	0.2936	1.0674	0.7675	0.026*	
H5B	0.5188	1.1733	0.8511	0.026*	
C12	0.2110 (5)	0.2269 (5)	1.4310 (3)	0.0216 (7)	
H12A	0.0667	0.1735	1.4136	0.032*	
H12B	0.2755	0.2930	1.5125	0.032*	
H12C	0.2521	0.1230	1.4159	0.032*	
C8	0.2582 (4)	0.6929 (4)	0.6429 (3)	0.0155 (6)	
H8	0.1827	0.5730	0.5896	0.019*	
C1	0.2312 (5)	0.5094 (4)	0.9097 (3)	0.0174 (6)	
H1	0.3183	0.6237	0.9659	0.021*	
C7	0.3432 (5)	0.8689 (5)	0.5959 (3)	0.0183 (6)	
H7A	0.2337	0.9002	0.5536	0.022*	
H7B	0.4068	0.8400	0.5402	0.022*	

### Atomic displacement parameters (Å^2^)

**Table:**

	*U*^11^	*U*^22^	*U*^33^	*U*^12^	*U*^13^	*U*^23^
Te	0.02669 (14)	0.01289 (13)	0.01485 (13)	0.00616 (9)	0.00697 (9)	0.00480 (8)
Cl	0.0278 (4)	0.0125 (3)	0.0153 (3)	0.0037 (3)	0.0050 (3)	0.0000 (3)
C10	0.0192 (14)	0.0146 (14)	0.0144 (14)	0.0061 (12)	0.0055 (12)	0.0032 (11)
C2	0.0138 (13)	0.0120 (13)	0.0167 (14)	0.0048 (11)	0.0054 (11)	0.0016 (11)
C4	0.0169 (14)	0.0158 (15)	0.0147 (14)	0.0026 (12)	0.0055 (11)	0.0026 (11)
C3	0.0147 (13)	0.0152 (14)	0.0144 (14)	0.0076 (11)	0.0061 (11)	0.0039 (11)
C6	0.0216 (15)	0.0183 (16)	0.0213 (16)	0.0039 (13)	0.0074 (13)	0.0084 (13)
C11	0.0221 (15)	0.0183 (15)	0.0153 (15)	0.0078 (13)	0.0049 (12)	0.0037 (12)
C9	0.0218 (15)	0.0181 (15)	0.0138 (14)	0.0061 (12)	0.0054 (12)	0.0055 (12)
C5	0.0265 (16)	0.0158 (15)	0.0193 (16)	0.0057 (13)	0.0064 (13)	0.0018 (12)
C12	0.0237 (16)	0.0266 (17)	0.0172 (15)	0.0112 (14)	0.0085 (13)	0.0073 (13)
C8	0.0165 (14)	0.0138 (14)	0.0151 (14)	0.0058 (11)	0.0043 (11)	0.0022 (11)
C1	0.0220 (15)	0.0123 (14)	0.0152 (14)	0.0047 (12)	0.0052 (12)	0.0036 (11)
C7	0.0214 (15)	0.0171 (15)	0.0148 (14)	0.0051 (12)	0.0069 (12)	0.0056 (12)

### Geometric parameters (Å, º)

**Table:**

Te—C1	2.077 (3)	C6—H6B	0.9900
Te—C9	2.148 (3)	C11—C12	1.526 (4)
Cl—C2	1.750 (3)	C11—H11A	0.9900
C10—C9	1.523 (4)	C11—H11B	0.9900
C10—C11	1.526 (4)	C9—H9A	0.9900
C10—H10A	0.9900	C9—H9B	0.9900
C10—H10B	0.9900	C5—H5A	0.9900
C2—C1	1.334 (4)	C5—H5B	0.9900
C2—C3	1.471 (4)	C12—H12A	0.9800
C4—C3	1.503 (4)	C12—H12B	0.9800
C4—C5	1.532 (5)	C12—H12C	0.9800
C4—H4A	0.9900	C8—C7	1.509 (4)
C4—H4B	0.9900	C8—H8	0.9500
C3—C8	1.338 (4)	C1—H1	0.9500
C6—C7	1.514 (4)	C7—H7A	0.9900
C6—C5	1.515 (5)	C7—H7B	0.9900
C6—H6A	0.9900		
			
C1—Te—C9	94.09 (12)	C10—C9—Te	110.3 (2)
C9—C10—C11	112.3 (3)	C10—C9—H9A	109.6
C9—C10—H10A	109.1	Te—C9—H9A	109.6
C11—C10—H10A	109.1	C10—C9—H9B	109.6
C9—C10—H10B	109.1	Te—C9—H9B	109.6
C11—C10—H10B	109.1	H9A—C9—H9B	108.1
H10A—C10—H10B	107.9	C6—C5—C4	110.7 (3)
C1—C2—C3	126.5 (3)	C6—C5—H5A	109.5
C1—C2—Cl	116.5 (2)	C4—C5—H5A	109.5
C3—C2—Cl	117.0 (2)	C6—C5—H5B	109.5
C3—C4—C5	112.1 (3)	C4—C5—H5B	109.5
C3—C4—H4A	109.2	H5A—C5—H5B	108.1
C5—C4—H4A	109.2	C11—C12—H12A	109.5
C3—C4—H4B	109.2	C11—C12—H12B	109.5
C5—C4—H4B	109.2	H12A—C12—H12B	109.5
H4A—C4—H4B	107.9	C11—C12—H12C	109.5
C8—C3—C2	122.8 (3)	H12A—C12—H12C	109.5
C8—C3—C4	121.6 (3)	H12B—C12—H12C	109.5
C2—C3—C4	115.7 (3)	C3—C8—C7	123.9 (3)
C7—C6—C5	110.2 (3)	C3—C8—H8	118.1
C7—C6—H6A	109.6	C7—C8—H8	118.1
C5—C6—H6A	109.6	C2—C1—Te	126.3 (2)
C7—C6—H6B	109.6	C2—C1—H1	116.9
C5—C6—H6B	109.6	Te—C1—H1	116.9
H6A—C6—H6B	108.1	C8—C7—C6	113.1 (3)
C12—C11—C10	111.6 (3)	C8—C7—H7A	109.0
C12—C11—H11A	109.3	C6—C7—H7A	109.0
C10—C11—H11A	109.3	C8—C7—H7B	109.0
C12—C11—H11B	109.3	C6—C7—H7B	109.0
C10—C11—H11B	109.3	H7A—C7—H7B	107.8
H11A—C11—H11B	108.0		
			
C1—C2—C3—C8	174.6 (3)	C7—C6—C5—C4	61.8 (4)
Cl—C2—C3—C8	−5.9 (4)	C3—C4—C5—C6	−48.2 (4)
C1—C2—C3—C4	−5.6 (4)	C2—C3—C8—C7	−179.0 (3)
Cl—C2—C3—C4	173.9 (2)	C4—C3—C8—C7	1.2 (5)
C5—C4—C3—C8	17.1 (4)	C3—C2—C1—Te	177.3 (2)
C5—C4—C3—C2	−162.7 (3)	Cl—C2—C1—Te	−2.2 (4)
C9—C10—C11—C12	−179.1 (3)	C9—Te—C1—C2	−177.3 (3)
C11—C10—C9—Te	179.5 (2)	C3—C8—C7—C6	12.0 (4)
C1—Te—C9—C10	179.5 (2)	C5—C6—C7—C8	−42.7 (4)

### Hydrogen-bond geometry (Å, º)

**Table:**

*D*—H···*A*	*D*—H	H···*A*	*D*···*A*	*D*—H···*A*
C8—H8···Cl	0.95	2.56	3.024 (3)	110

## References

[bb1] Altomare, A., Burla, M. C., Camalli, M., Cascarano, G. L., Giacovazzo, C., Guagliardi, A., Moliterni, A. G. G., Polidori, G. & Spagna, R. (1999). *J. Appl. Cryst.* **32**, 115–119.

[bb2] Barrientos-Astigarraga, R. E., Castelani, P., Sumida, C. Y., Zukerman-Schpector, J. & Comasseto, J. V. (2002). *Tetrahedron*, **58**, 1051–1059.

[bb3] Bondi, A. (1964). *J. Phys. Chem.* **68**, 441–452.

[bb4] Brandenburg, K. (2006). *DIAMOND* Crystal Impact GbR, Bonn, Germany.

[bb5] ChemAxon (2010). *MarvinSketch.* http://www.chemaxon.com.

[bb6] Cremer, D. & Pople, J. A. (1975). *J. Am. Chem. Soc.* **97**, 1354–1358.

[bb7] Farrugia, L. J. (1997). *J. Appl. Cryst.* **30**, 565.

[bb8] Guadagnin, R. C., Suganuma, C. A., Singh, F. V., Vieira, A. S., Cella, R. & Stefani, H. A. (2008). *Tetrahedron Lett.* **49**, 4713–4716.

[bb9] Higashi, T. (1995). *ABSCOR* Rigaku Corporation, Tokyo, Japan.

[bb10] Molecular Structure Corporation & Rigaku (2005). *CrystalClear* MSC, The Woodlands, Texas, USA, and Rigaku Corporation, Tokyo, Japan.

[bb11] Sheldrick, G. M. (2008). *Acta Cryst.* A**64**, 112–122. 10.1107/S0108767307043930 18156677

[bb12] Westrip, S. P. (2010). *J. Appl. Cryst.* **43**, 920–925.

[bb13] Zeni, G., Chieffi, A., Cunha, R. L. O. R., Zukerman-Schpector, J., Stefani, H. A. & Comasseto, J. V. (1999). *Organometallics*, **18**, 803–806.

